# Photoswitchable molecular tweezers: isomerization to control substrate binding, and what about *vice versa*?

**DOI:** 10.1039/d2cc04329g

**Published:** 2022-09-15

**Authors:** Sander J. Wezenberg

**Affiliations:** Leiden Institute of Chemistry, Leiden University, Einsteinweg 55 2333 CC Leiden The Netherlands s.j.wezenberg@lic.leidenuniv.nl

## Abstract

The linkage of two identical binding motifs by a molecular photoswitch has proven to be a straightforward and versatile strategy to control substrate binding affinity by light. Stimulus control of binding properties in artificial receptors is partly inspired by the dynamic behavior of proteins and is highly attractive as it could, for example, improve extraction processes and allow (de)activation of membrane transport on demand. This feature article summarizes the development and design principles of molecular tweezers containing a molecular photoswitch as the core unit. Besides the control of binding affinity by isomerization, the effect of substrate binding on the isomerization behavior is discussed where data is available. While the latter often receives less attention, it could be of benefit in the future creation of multi-stimuli-controlled molecular switching and machine-like systems.

## Abstract 2

Functionalization of molecular photoswitches with binding entities offers control of affinity, while isomerization properties can be influenced by bound substrate.

## Graphical abstract

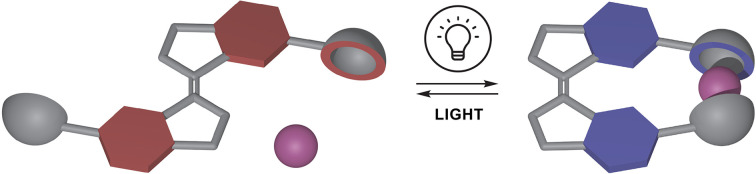

## 1. Introduction

**Scheme 1:**
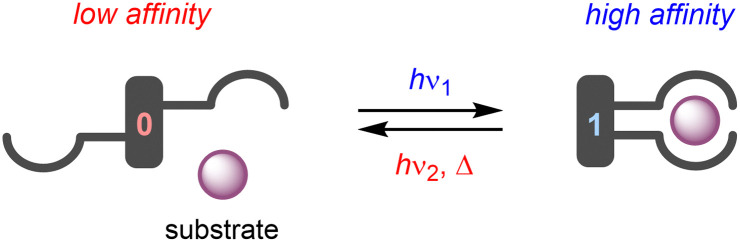
General design of photoswitchable molecular tweezers.

**Scheme 2:**
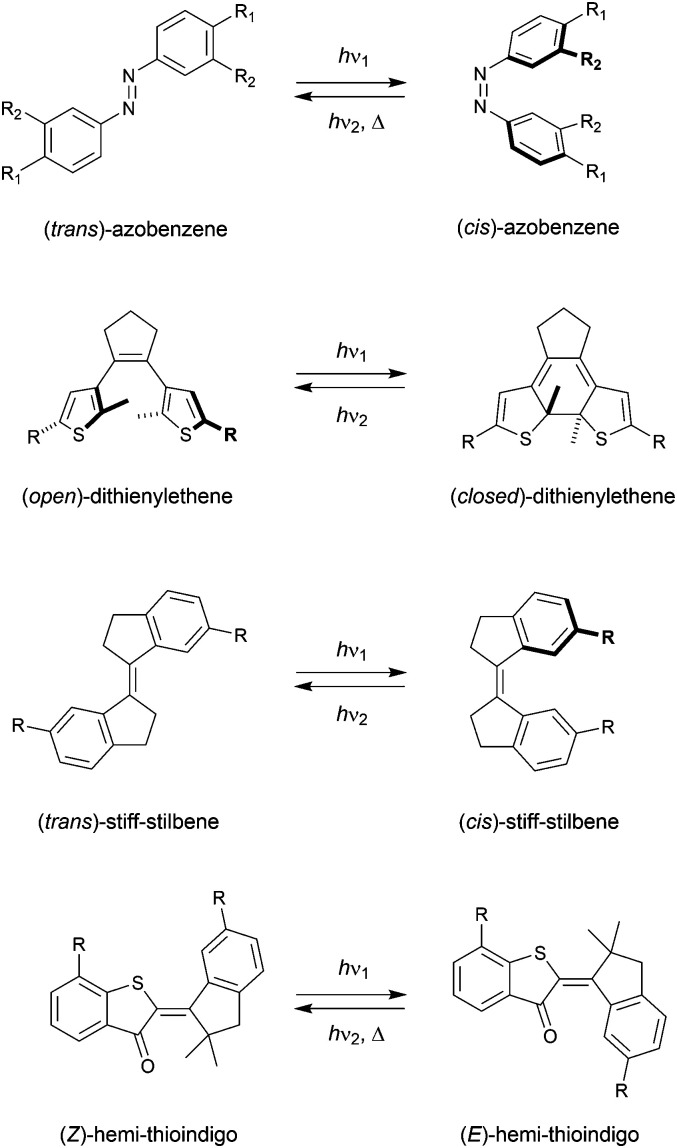
Molecular photoswitches used as platforms for molecular tweezers.

The term “molecular tweezer” was first coined by Whitlock to describe two aromatic moieties (caffeine) linked by a rigid diyne unit, able to bind aromatic guests *via* π–π interactions in a sandwich-type complex.^1^ The binding affinities were found to greatly exceed that of the individual aromatic components due to the cooperative nature. In general, any binding motif can be chosen in such a design, however, a critical element is the spacer by which they are connected. This spacer needs to be fairly rigid and give the correct preorganization (*i.e.*, separation and orientation) of binding entities.^2^ The incorporation of a molecular photoswitch as the central unit has enabled dynamic control of substrate binding (Scheme 1). Here, in one of the photoaddressable states, the binding moieties are brought together to simultaneously bind the substrate (high affinity), while in the other the same binding mode is not possible due to steric constraints (low affinity). The group of Shinkai pioneered this field by developing photoresponsive crown ethers based on azobenzene.^3,4^ This concept has later been extended to the attachment of various binding motifs and the utilization of other molecular photoswitches, *i.e.*, dithienylethene,^5^ stiff-stilbene,^6^ and hemi-thioindigo^7^ (Scheme 2). The mode of operation of these photoswitches has been summarized in other accounts.^8^ The emphasis here lies on their suitability for use as backbone of photoswitchable molecular tweezers, which is discussed per class of photoswitch in each of the sections.

It should be noted that there is large interest in controlling binding properties of artificial receptors by external stimuli and the use of light has proven particularly promising towards this goal.^9^ Part of the work in this field is inspired by the dynamic functions of proteins, such as the regulation of solute transport across the cell membrane. Imitation of this in synthetic systems may result in physiological tools and therapeutic agents^8,10^ to respectively study and treat diseases that have been associated to malfunctioning protein transporters [*e.g.*, mutations in the gene encoding for cystic fibrosis transmembrane conductance regulator (CFTR) protein leads to dysregulation of chloride translocation]. In addition, stimuli-responsive receptors may be used to improve the efficiency of chemical extraction processes as separation from the extracted substrate and recovery is facilitated by switching to the low-affinity form.^11^ Multiple approaches have therefore been developed to control binding affinity by light, with molecular tweezers being among the most straightforward ones. Other successful examples have been based on foldamers,^12^ macrocycles,^13^ capsules and cages,^14^ which were reviewed earlier by us and others.^15^ To the best of our knowledge, a comprehensive discussion on the design principles of photoswitchable molecular tweezers has not been reported in recent years.

Where the main focus is on demonstrating reversible switching in combinations with distinct binding affinity for each of the interchangeable isomers, less attention is often devoted to the effect of substrate binding on the isomerization behavior itself. Isomerization studies are not always performed in presence of the guest species, but it is recommendable to do so, since it can significantly alter or even impede switching processes. This does not necessarily have to be a drawback. It could be used to one's advantage, for example, to create multi-stimuli responsive (gated) molecular switching and machine-like systems with high levels of complexity and sophistication.^16^ Hence, the influence of substrate binding on the isomerization properties is worth a more detailed discussion. In this feature article, in first place, (symmetrical) molecular tweezer designs containing different photoswitchable cores are discussed, including stiff-stilbene based anion receptors developed by our group. Experimental results regarding changes in thermal and photoisomerization behavior upon substrate binding are highlighted where available, and placed into a broader context.

## 2. Azobenzene

The first photoswitchable molecular tweezers developed were based on azobenzene, which has been widely used to construct molecular devices and functional materials.^4^ Where originally UV light was needed for isomerization from the more stable *trans* isomer to the *cis* isomer, various functionalization strategies are now available to shift the excitation wavelength into the visible-light region.^17^ The reverse isomerization back to the *trans* isomer usually occurs thermally in the dark, while it can be accelerated by white light irradiation. Thermal back isomerization impedes isolation and individual characterization of the *cis* isomer and therefore, binding constants are often reported as average values for the photostationary state (PSS) *cis*/*trans* mixtures. Owing to the pronounced geometrical change upon isomerization, azobenzene is a suitable scaffold for developing photoswitchable molecular tweezers. However, *trans*-to-*cis* isomerization brings about an increase in dipole moment and water solubility, which may cause undesired partial transfer to the aqueous phase during extraction and transport studies.^18^

### 2.1 (Alkali) metal ion complexation

Cation complexation to molecular hosts has been studied since the dawn of host-guest chemistry, which was marked by Pedersen's discovery of crown ethers.^19^ The ability of antibiotics to transport cations across cellular membranes inspired chemists to synthesize a diverse range of ionophore mimics that exhibit remarkable selectivities and transport properties.^20^ Shinkai and Manabe pioneered the development of photoresponsive cation receptors,^3^ among which are molecular tweezers, with the goal of controlling extraction and transport properties by light.

**Scheme 3:**
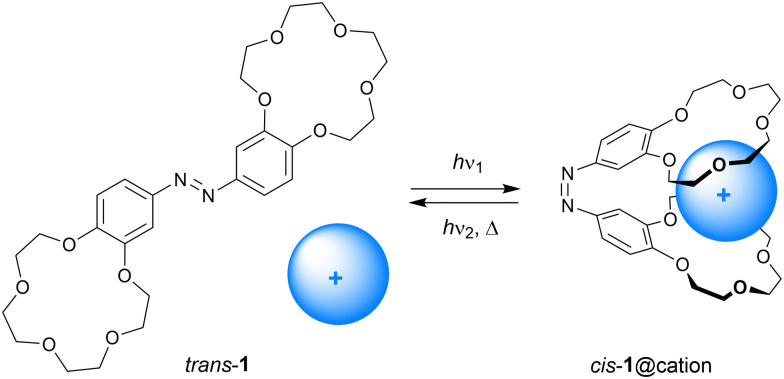
Photostimulated cation (*i.e.*, K^+^, Rb^+^, Cs^+^) binding by azobis(benzo-15-crown-5).

In 1980, Shinkai *et al.* described azobenzene bridged bis(benzo-15-crown-5) 1 shown in Scheme 3.^18^ Irradiation of this compound in *o*-dichlorobenzene using a Hg-lamp afforded an equilibrium (*cis*/*trans*) ratio of approximately 51 : 49 and back isomerization from the *cis* to the *trans* isomer occurred relatively fast in the dark (*t*_1/2_ = 10.3 min at 30 °C). The binding ability of this receptor was studied by extraction of an aqueous solution of alkali metal salts of methyl orange to *o*-dichlorobenzene. It had been established earlier for benzo-15-crown-5 that alkali metal cations that exactly fit the ring size of the crown ether form 1 : 1 inclusion complexes, whereas metal cations with larger atomic radii are sandwiched by two crown ethers in a 2 : 1 complex.^19^ This difference in binding stoichiometry gives rise to contrasting selectivities for the *cis* and *trans* isomers of 1. That is, *trans*-1 extracted Na^+^ more efficiently than *cis*-1 (Ex_*trans*_/Ex_*cis*_ = 5.6), but the larger K^+^ ion was more efficiently extracted with *cis*-1 than with *trans*-1 (Ex_*cis*_/Ex_*trans*_ = 42.5). Also, the relatively large Rb^+^ and Cs^+^ ions were the most efficiently extracted by the *cis* isomer. The reason for this difference in extraction ability is that in the *cis* form both benzo-15-crown-5 moieties bind the larger cations in a cooperative manner.

Interestingly, the *cis*/*trans* ratio at the PSS significantly improved by the addition of Rb^+^ and Cs^+^ ions (98 : 2 and 89 : 11, respectively) while, on the other hand, the rate of thermal *cis*-to-*trans* isomerization was suppressed.^21^ Although electronic effects were considered, this observation was ascribed to stabilization of the *cis* isomer as a result of the strong 1 : 1 sandwich-type complexation with the cation. Nevertheless, the *cis*-to-*trans* isomerization step could be accelerated by irradiation with visible light.^22^

The effect of photoisomerization on the rate of cation transport through a liquid *o*-dichlorobenzene membrane was examined in a U-tube using different counteranions.^21,22^ For K^+^ picrate, for example, UV irradiation lowered the rate of transport by 1.9 fold, most likely because cation release from the *cis* isomer becomes rate-limiting. Hence, the transport rate is significantly increased by alternate irradiation with UV (*λ* > 360 nm) and visible (*λ* > 460 nm) light, and was enhanced further by exposing the feeding phase to UV and the receiving phase to visible light.

In a later stage, the group of Shinkai combined a benzo-18-crown-6 analogue with diammonium cations, which allowed switching between polymeric and pseudocyclic complexes.^23^ In addition to alkali metals, ammonium cations are well known to bind to the crown ether motif.^19^ When hexamethylene diammonium tosylate was added, the *cis* isomer formed a (pseudocyclic) 1 : 1 complex. However, the distance between the ammonium groups of this guest turned out to be too short to bridge the 18-crown-6 moieties in the *trans* isomer and, as a result, a polymeric structure was obtained. The two types of complexes could be interconverted by irradiation with UV and visible light as was reflected in viscosity and conductance measurements, which were carried out in mixtures of *o*-dichlorobenzene and 1-butanol. The viscosity of the solution decreased upon irradiation with UV light and was regained by subsequent irradiation with visible light. Conversely, the conductance increased upon exposure to UV light and gradually decreased when using visible light.

Toward control of Zn(ii) ion binding, Erlanger and co-workers equipped azobenzene with two iminodiacetic acid groups.^24^ The *trans* isomer did not bind Zn(ii) ions but the *cis* isomer, which was generated in about 80% yield by 320 nm irradiation, did with an association constant of *K*_a_ = 1.1 × 10^5^ M^−1^ in H_2_O. The PSS_320_ mixture was stable for many days in the dark, while exposure to white light gave an equilibrium mixture (*cis*/*trans*) of 20 : 80.

**Fig. 1:**
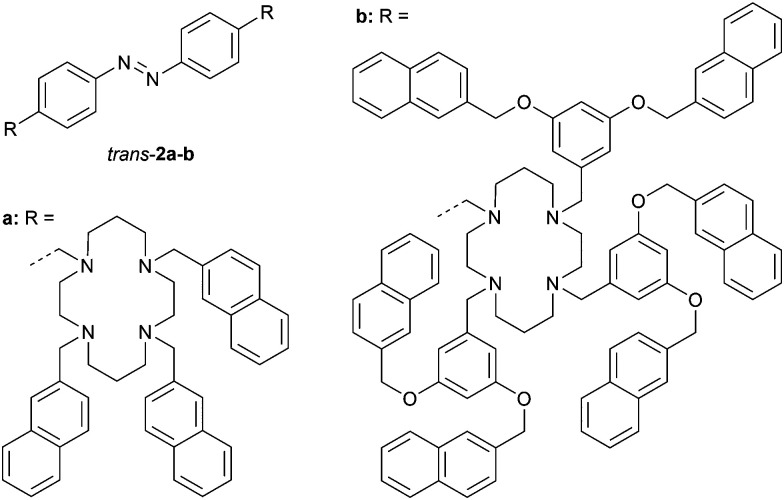
Naphthalene-functionalized and cyclam-appended azobenzene derivatives 2a–b.

Alternatively, Ceroni and co-workers functionalized azobenzene with cyclam metal-coordinating motifs having naphthalene chromophores attached (2a–b, Fig. 1).^25^ The naphthalene chromophores can be excited by 275 nm light and exhibit luminescence, however, the emission quantum yields were significantly reduced by the attachment to azobenzene, suggesting quenching. Isomerization from the *trans* to *cis* isomer was triggered by 365 nm light, where only the azobenzene unit absorbs, and afforded 95 : 5 and 93 : 7 (*cis*/*trans*) ratios at the PSS for 2a and 2b, respectively. The *cis* isomers could also be generated by irradiation with 275 nm, at which the absorption of the naphthalene units is dominant (>95%), indicating energy transfer from naphthalene to the *trans*-azobenzene core. Irradiation of the PSS_365_ mixtures with 436 nm light led to the reverse *cis*-to-*trans* isomerization process.

Titrations experiments with Zn(ii) ions in MeCN/CH_2_Cl_2_ (1 : 1, v/v) revealed strong complexation with *cis*-2a in a 1 : 1 stoichiometry (*K*_a,1_ = 1 × 10^8^ M^−1^), while complexation with *trans*-2a was weaker and occurred in a 1 : 2 fashion (*K*_a,1_ = 7 × 10^7^ M^−1^ and *K*_a,2_ = 5 × 10^6^ M^−1^). Similar results were obtained when compound 2b was used. Photoirradiation in the presence of excess Zn(ii) ions (6 equiv.) revealed an increase in the content of *trans* isomers at the PSS (90 : 10 for 2a and 87 : 13 for 2b, *cis*/*trans*) as a result of lowering the quantum yields for *trans*-to-*cis* isomerization. The binding properties of Cu(ii) ions were similar, however, their complexation impeded photosensitization and further reduced the quantum yields of photoisomerization, consistent with energy/electron transfer to the ligated Cu(ii) ions. Beside the demonstrated control over ion binding by light, the ion itself could thus be used as a stimulus to influence the photophysical properties of the system.

### 2.2 Anion binding and transport

Driven by the important role of anions in biological and environmental processes, many artificial receptors for anionic substrates have been developed.^26^ These receptors have been applied in sensing of analytes,^27^ extraction of pollutants,^28^ and transport of anions across lipid bilayer membranes.^29^ Toward the development of anion-binding molecular tweezers, mostly hydrogen bond donating (thio)urea groups have been used.^30^

**Fig. 2:**
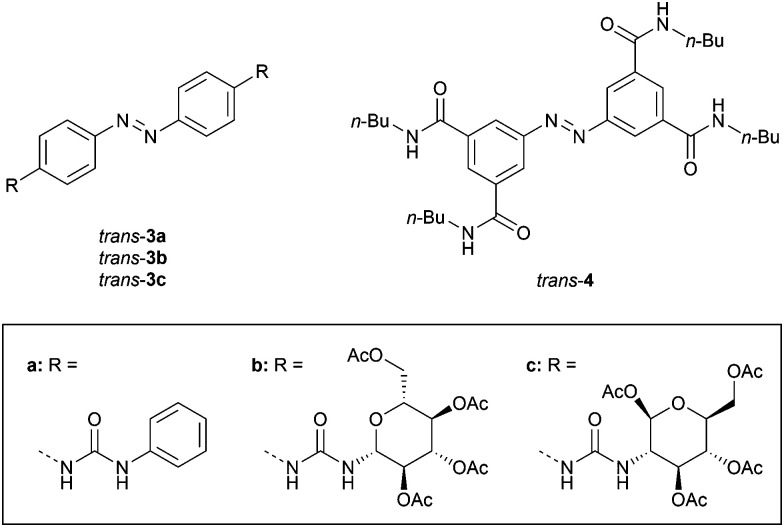
Azobenzene-based tweezers developed by Dąbrowa and Jurczak.

Dąbrowa and Jurczak developed bis-urea substituted azobenzene 3a (Fig. 2), of which the *cis* isomer was generated by UV irradiation (*λ* = 368 nm) and the *trans* isomer was regained by using visible light (*λ* = 410 nm).^31^ Somewhat surprisingly, the binding affinity of benzoate to *cis*-3a (*K*_a,1_ = 2.3 × 10^2^ M^−1^) was about 3–4 times lower than to *trans*-3a 1 : 2 (*K*_a,1_ = 9.9 × 10^2^ M^−1^), as was determined in DMSO/0.5%H_2_O. This weaker binding was partially explained by steric repulsion between the phenyl groups of benzoate and the *cis* isomer. Interestingly, the urea functionality in the *para*-position bathochromically shifted the π–π* absorption band, and significantly reduced the half-life of *cis*-3a (*t*_1/2_ = 1.8 h in DMSO/0.5%H_2_O) compared with unsubstituted azobenzene (*t*_1/2_ = 214 h in DMSO/0.5%H_2_O), Moreover, these effects were amplified in the presence of anions, with the lowest half-life observed for acetate (*t*_1/2_ = 8 min), which was among the most basic anions used in the series and has a high structural complementarity to urea.^30^ The increase in the thermal isomerization rate was attributed to transfer of electron density from the anion to the azobenzene N=N bond region, causing an increase in lone pair repulsion. This observation is in line with that of Shinkai, who found the exact opposite trend on the rate of thermal *cis*-to-*trans* isomerization upon strong binding of cations to crown ether-appended azobenzene. In that case, however, this phenomenon was ascribed to the bridging of the two crown ethers by the cation in the *cis* isomer, rather than electron-withdrawing effects, since the effect was only noted for the largest alkali metal cations able to give 1 : 1 complexes.

The same authors later exchanged the phenyl substituents for carbohydrates to control chiral discrimination of amino carboxylates (3b–c, Fig. 2).^32^ The *cis* isomers were again obtained by 368 nm irradiation and their half-lives were similar to the phenyl-substituted analogue (*t*_1/2_ = 1.1 and 2.4 h for 3b and 3c, respectively). Also here, the binding of benzoate to the *trans* isomer was stronger than to the *cis* isomer (*K*_*trans*_/*K*_*cis*_ = 1.6 for 3b and 3.0 for 3c), while for acetate no significant difference in affinity between isomers was noted, which supports the suggested steric hindrance with the phenyl ring of benzoate in *cis*-3a–c. In addition, chiral phenylalanine and tryptophan carboxylate salts were bound 2–3 times stronger by the *trans* than the *cis* isomer, with an overall selectivity towards the d-enantiomers. The thermal *cis*-to-*trans* isomerization step was again accelerated by addition of acetate, and proceeded the fastest in the presence of the more strongly binding enantiomers of the chiral guests.

In a later stage, Dąbrowa *et al.* reported azobenzene derivative 4 (Fig. 2), which has four hydrogen bond-donating amide substituents in the *meta*-positions.^33^ In this case, the thermal stability of the *cis* isomer – produced in 22% yield by 368 nm irradiation – was unaffected. Now, the close positioning of the amide groups in the *cis* isomer did favor the binding of anions over the *trans* isomer (*K*_*cis*_/*K*_*trans*_ = 2–3). The receptor was particularly selective for dihydrogen phosphate (*K*_a,1_ = 4.4 × 10^2^ M^−1^ and 2.0 × 10^2^ M^−1^ for *cis*-4 and *trans*-4 in DMSO/0.5%H_2_O, respectively). This anion was expected to have better size and shape complementarity than the more basic (and therefore usually stronger binding) acetate and benzoate anions.

**Scheme 4:**
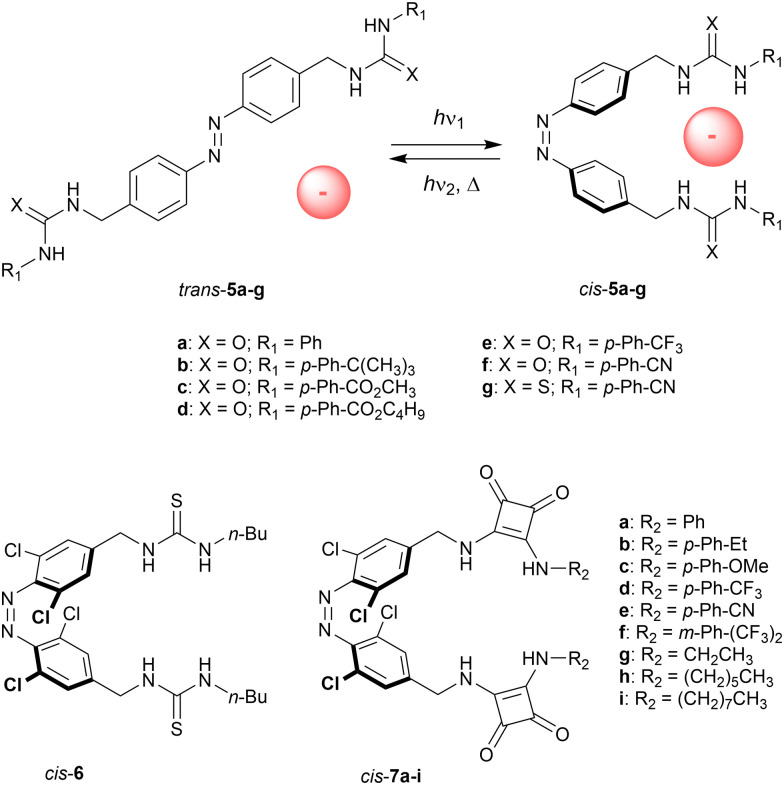
Photoswitchable bis-(thio)urea and bis-squaramide receptors/transporters.

The group of Jeong installed (thio)urea substituents in the benzylic positions (5a–g, Scheme 4).^34^ UV irradiation (*λ* = 365 nm) afforded the *cis* isomers (90–96% yield). Titrations with chloride revealed *K*_*cis*_/*K*_*trans*_ ratios between 5–10, illustrating that only in the *cis* isomer both (thio)ureas can be involved in hydrogen bonding to a single anion. For both isomers, electron-withdrawing substituents enhanced the chloride binding affinities, which is expected based on the higher NH proton acidities.

Using a Cl^−^/NO_3_^−^ ion-selective electrode (ISE) exchange assay and unilamellar POPC vesicles, the capability of 5a–g to transport chloride ions across the bilayer membrane was assessed. These experiments revealed negligible activity for the *trans* isomers, whereas the *cis* isomers showed moderate to good activities, with the best performance observed for *p*-cyano-substituted phenyl(thio)urea 5g (EC_50_ = 0.19 mol% to lipid). Hence, this compound was chosen to demonstrate activation of chloride transport *in situ*. That is, by starting with *trans*-5g and irradiation of the vesicles with 365 nm light, transport was activated as a result of isomerization to *cis*-5g. The higher activity for the *cis* isomers with respect to the *trans* isomers was partly attributed to the stronger chloride binding ability, while an additional explanation was sought in altered mobility and partitioning in the membrane as a result of the change in structure and dipole.

A variant with four *ortho*-chloro substituents was later reported by Wang and co-workers (6, Scheme 4).^35^ This type of substitution is known to cause a bathochromic shift of the excitation wavelength^17^ and hence, *cis*-6 could be produced by irradiation with red light giving a PSS ratio of 31 : 69 (*cis*/*trans*). This receptor was shown to bind bis-carboxylate guests having different alkyl bridge lengths in a 1 : 1 stoichiometry and, while azelate bound the strongest to both isomers, the shorter adipate had a higher affinity for the *cis* than the *trans* isomer (*K*_a_ = 3.1 × 10^3^ M^−1^ for PSS_red_ and 1.5 × 10^3^ M^−1^ for *trans*-6 in DMSO). Presumably, the improved binding to the *cis* isomer stems from a better match between the thiourea-to-thiourea distance and the length of the adipate bis-anion.

More recently, Kerckhoffs and Langton changed the (thio)urea motifs for squaramide (7a–i, Scheme 4),^36^ which has superior anion binding and transport properties.^37^ When the NH-Boc protected precursor was irradiated with red light (*λ* = 625 nm), 80% of *trans* isomer was converted to *cis* isomer. In accordance with the work of Jeong, titrations with chloride in DMSO revealed the highest association constants for the *cis* compounds (*K*_*cis*_/*K*_*trans*_ ∼ 3). Here, transport across POPC bilayers was initially studied with an HPTS fluorescence assay, which showed significantly higher activity for the *cis* than the *trans* isomers (*e.g.*, 8-fold in case of 7a, EC_50(PSS)_ = 0.07 mol% to lipid). As the difference in activity is larger than that in binding affinity, it was reasoned that improved mobility and encapsulation ability upon *trans*-to-*cis* isomerization plays an important additional role in the transport enhancement. Using an ISE Cl^−^/NO_3_^−^ exchange assay, the authors were able to demonstrate reversible photocontrol of transport activity for the first time. After *trans*-7a was added as DMSO solution to POPC vesicles, transport was activated by irradiation with 625 nm light (∼50% of activity relative to a pre-irradiated sample), and almost fully deactivated by subsequent irradiation with 455 nm light.

In a following study, the group of Langton synthesized a *meta*-substituted variant of 7a, introduced a more flexible C2-linker between azobenzene and the squaramide binding motifs, and prepared an analogue with tetra-*ortho*-fluoro substituents.^38^ Overall, the transport activity of these derivatives was higher, but the difference in binding and transport behavior between isomers was less than for 7a.

**Fig. 3:**
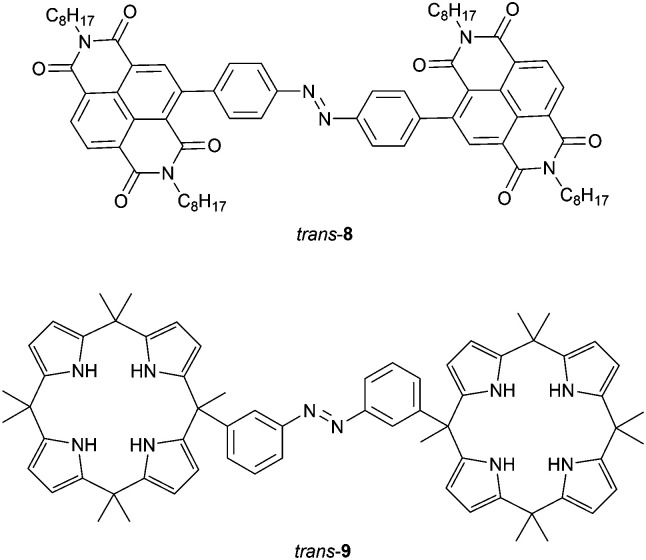
Naphthalenediimide- and calix[4]arene-appended azobenzene receptors.

Bandyopadhyay and Bhosale demonstrated that fluoride binding to naphthalenediimide (NDI) substituted 8 (Fig. 3) strongly affects its isomerization behavior.^39^ By 366 nm irradiation, *cis*-8 could be produced, albeit that photoconversion was slow. This isomer turned out to have a high thermal stability but, nevertheless, the reverse *cis*-to-*trans* isomerization could be induced by visible light (*λ* = 500 nm). It had been reported before that fluoride binding *via* anion–π interactions can lead to formation of a radical anionic NDI˙^−^ and dianionic NDI^2−^ species.^40^ Irradiation of *trans*-8 with UV light in the presence of fluoride (1 equiv.) gave full conversion to the radical anion of the *cis* isomer, in which the fluoride ion is bridged between the NDI substituents. Further addition of fluoride (≥2 equiv.) afforded the dianionic species and interestingly, both anion-complexed *cis* forms did not revert to the *trans* form by visible light irradiation neither thermally, unless an oxidant (NOBF_4_) was added first to dissociate the sandwich-type complex.

Cafeo *et al.* connected two macrocyclic calix[4]pyrrole^41^ receptors with azobenzene (9, Fig. 3) and, similar to Wang and co-workers, studied the binding of bis-carboxylates with different alkyl spacer lengths.^42^ While in DMSO solution the *cis* isomer, which was generated by 365 nm irradiation, had the highest affinity for the shorter succinate (*K*_a_ = 5.3 × 10^5^ M^−1^; *K*_*cis*_/*K*_*trans*_ = 83), the *trans* isomer exhibited the strongest binding toward the longer adipate anion (*K*_a_ = 8.6 × 10^4^ M^−1^; and *K*_*cis*_/*K*_*trans*_ = 0.26). The binding of bis-carboxylates was found to affect both photochemical and thermal isomerization properties. First, the rate of light-induced *trans*-to-*cis* isomerization decreased with increasing association constant for *trans*-9. Second, the rate of thermal *cis*-to-*trans* isomerization changed, however, without direct correlation to the binding strength. Although in the presence of succinate, which bound the strongest to *cis*-9, thermal isomerization was the slowest, the rate was actually increased relative to the free receptor for the other bis-carboxylate guests. The authors speculated that guest binding can have two different and opposing effects: (i) bridging of the binding motifs slows down thermal isomerization because of steric reasons and (ii) binding of anions speeds up the process due to electron density transfer to the azobenzene core. If the latter would be the decisive factor for all guests used, except for succinate, it may explain the observations in this study. However, such electronic effects were previously only observed for anion binding to *para*-substituted azobenzene and excluded in the tetra-*meta*-amide substituted azobenzene receptor reported by Dąbrowa *et al.*

### 2.3 Catching neutral substrates

In addition to positively and negatively charged species, a small number of photoswitchable molecular tweezers have been developed to bind non-ionic substrates, most notably by taking benefit from solvophobic effects or π–π stacking interactions.

For example, azobenzene was functionalized with two β-cyclodextrin units by Aoyagi *et al.*^43^ The resulting compound was irradiated with 320–380 nm light to produce the *cis* isomer in 66% yield, having a half-life of 34 h at 25 °C. This *cis* isomer exhibited much stronger Circular Dichroism (CD) absorption than the starting *trans* isomer and hence, CD spectroscopy was used to study the influence of guest binding on the thermal isomerization rate. This rate was expected to decrease in the presence of a guest that would simultaneously bind the two β-cyclodextrin cavitands, however, the potential guest molecules that were used (*e.g.*, 1-adamantanol and ursodeoxycholic acid) did not show any noticeable effect.

In a related study, the group of Rebek Jr. bridged deep resorcinarene cavitands with an azobenzene spacer.^44^ The PSS ratio obtained upon 365 nm irradiation was determined as 62 : 38 (*cis*/*trans*) and changed when monotopic and ditopic adamantane guests were added. In the presence of *N*-butyladamantane-1-carboxamide, for example, a ratio of 71 : 29 (*cis*/*trans*) was observed. Furthermore, the *trans* isomer could be regenerated upon heating, and by addition of ditopic adamantane guests that fit this isomer better than the *cis* one, the rate of *cis*-to-*trans* isomerization was enhanced.

**Fig. 4:**
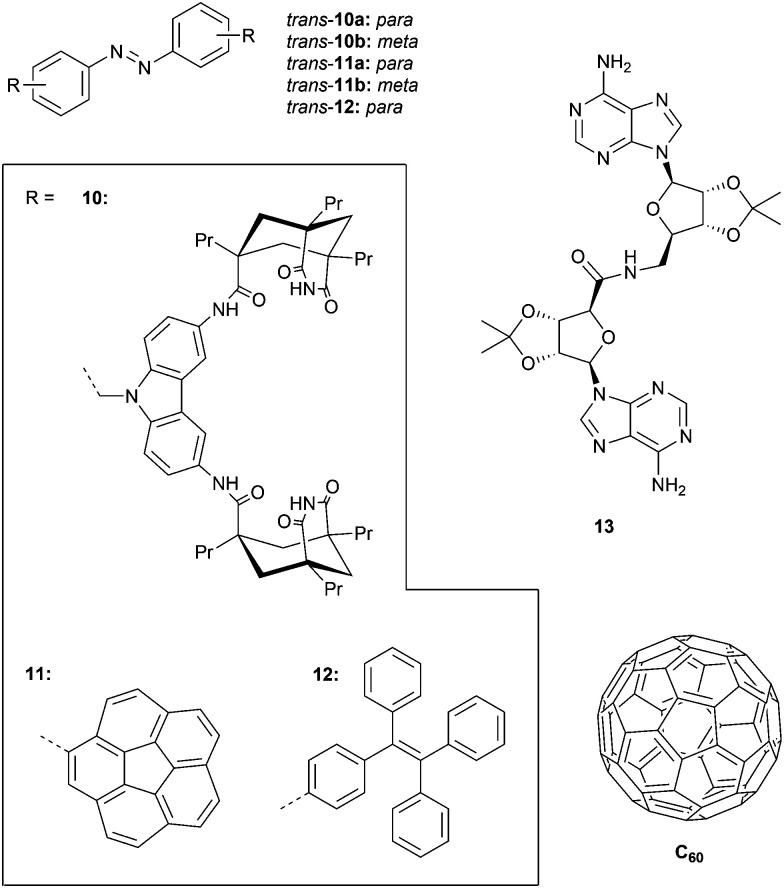
Azobenzene-based receptors 10–12 and the neutral substrates 13 and C_60_ they bind.

In an earlier example, the same group developed tweezers 10a–b bearing two adenine binding sites (Fig. 4).^45^ Irradiation with 366 nm light was shown to result in photoequilibrium mixtures containing only 50% of *cis* isomer, which is much lower than that obtained for methyl-substituted azobenzene (90–94%). The lower conversion towards the *cis* isomer was ascribed to absorption overlap with the carbazole chromophore, which could give energy transfer to the *cis* isomer causing back isomerization to the *trans* isomer. Furthermore, the reverse *cis*-to-*trans* isomerization could be triggered intentionally by visible light irradiation (*λ* > 400 nm) affording *cis*/*trans* ratios at the PSS of 30 : 70 and 25 : 75 for 10a and 10b, respectively, being unaffected by the carbazole substituents.

Compounds 10a–b were used as catalysts to photocontrol a coupling reaction giving product 13 (Fig. 4) and as anticipated, irradiation of the *trans* isomers with 366 nm light resulted in a reaction rate enhancement (of 10-fold for 10a). In addition, an inhibitory effect of the product on catalysis was observed, suggesting strong and simultaneous binding by both adenine groups. This observation motivated the authors to study its effect on the *cis*-to-*trans* thermal isomerization rate. Addition of one equivalent of 13 decreased this rate, whereas in the presence of larger amounts an increase was noted, most likely reflecting the transition from a 1 : 1 to a 1 : 2 complex.

With the goal of binding fullerenes C_60_ and C_70_, the group of Álvarez developed corannulene derivatives 11a–b (Fig. 4).^46^ Photoisomerization using 365 nm light afforded *cis*/*trans* ratios of around 20 : 80 at the PSS and, upon heating at 80 °C for 30 min, 95% of the *trans* isomer was recovered. The conversion toward the *cis* isomer was improved by using 380 nm instead of 365 nm light as the absorption maximum appeared to be red-shifted compared to regular azobenzene because of corannulene substitution. For compound 11a, it was found (in toluene solution) that the *trans* isomer is not able to associate with C_60_ and C_70_, but that the *cis* isomer does with estimated equilibrium constants of around *K*_a_ ∼ 2.5 × 10^3^ M^−1^. In contrast, *trans*-11b did bind C_60_ and C_70_ (*K*_a_ = 5.0 × 10^2^ M^−1^ and 8.3 × 10^2^ M^−1^, respectively) and showed minor differences in binding behavior with *cis*-11b (*K*_a_ = 6.7 × 10^2^ M^−1^ and 2.5 × 10^2^ M^−1^, respectively).

Likewise, Bhosale and Bandyopadhyay achieved photocontrol over C_60_ binding by using the tetraphenylethene (TPE) substituted 12 (Fig. 4).^47^ They used 254 nm irradiation to produce the *cis* isomer, affording a 3 : 1 (*cis*/*trans*) ratio when the PSS was reached, and the reverse *cis*-to-*trans* isomerization was achieved using visible light (*λ* > 400 nm). The stability constant for *cis*-12⊂C_60_ (*K*_a_ = 4.0 × 10^4^ M^−1^), determined in CS_2_, was more than 20 times larger than for *trans*-12⊂C_60_ (*K*_a_ = 1.8 × 10^3^ M^−1^). When the *cis* isomer in the presence of C_60_ was exposed to visible light (*λ* > 490 nm), quantitative conversion to the *trans* isomer was achieved, with concomitant *in situ* guest release.

### 2.4 Influence on isomerization behavior

To briefly summarize, in the examples where photoirradiation in presence of the guest species was described, the PSS ratios were found to be more favored toward the stronger binding *cis* isomer. On the other hand, the influence of guest binding on the rate of thermal isomerization differed. In general, bridging of the binding motifs by the guest in the *cis* isomer was found to increase the half-life, which has been ascribed to stabilization in the 1 : 1 sandwich-type complex. Nevertheless, electronic effects could also play an important role as was demonstrated for the binding of anionic species to urea-functionalized azobenzenes. An acceleration of *cis*-to-*trans* isomerization was observed in that case, which was explained by transfer of electron density from the substrate to the central N=N bond region, leading to increased lone pair repulsion. The binding of anions could thus have opposing effects, whereas for cations it may be challenging to dissect the individual contributions of complex stabilization and electronic effects to an observed increase in *cis*-to-*trans* isomerization rate.

## 3. Dithienylethene

The advantages of choosing dithienylethene over azobenzene as the photoswitchable core are its excellent fatigue resistance and the high thermal stability of the photogenerated isomer.^5^ However, as the change in geometry upon photoisomerization is less pronounced, differences measured in binding affinity and extraction ability between interconvertible isomers are relatively small. Furthermore, for dithienylethene-based molecular tweezers, it is the starting *open* form that exhibits the highest binding affinity owing to its ability to form a sandwich-type complex with a suitable guest molecule. Photocyclization to the *closed* form may lead to guest ejection as a result of rigidification of the molecular geometry.

### 3.1 Cation binding and quantum yield changes

**Scheme 5:**
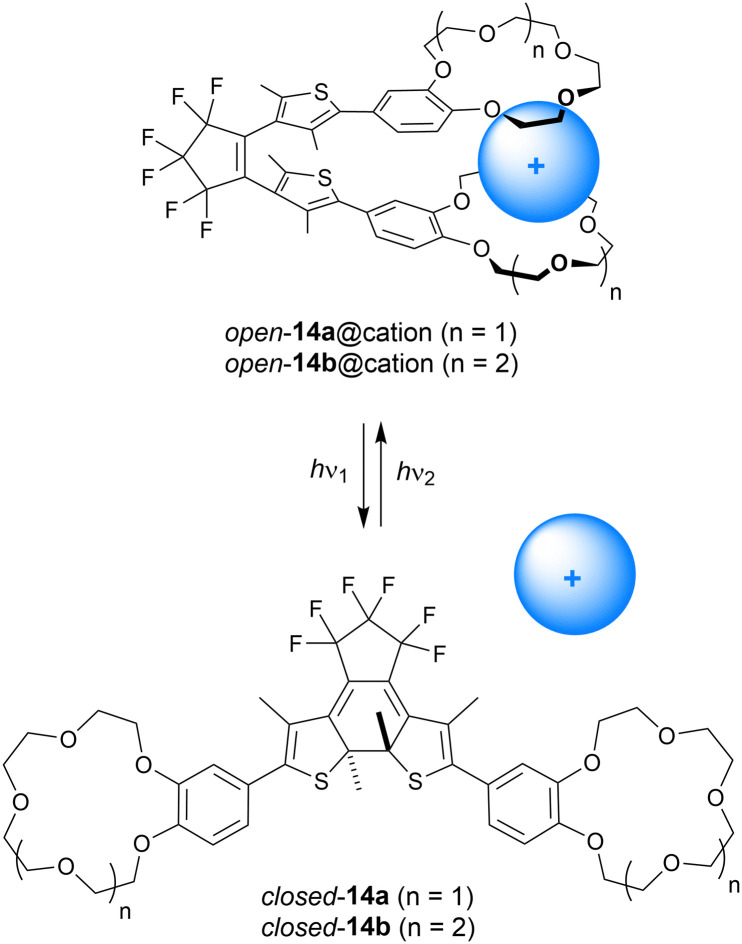
Modulation of alkali metal cation binding to dithienylethenes 14a–b containing benzocrown substituents. The open-ring form is drawn in the parallel conformation.

Where the group of Shinkai linked two crown ether moieties by azobenzene, Takeshita and Irie used dithienylethene as the photoswitchable scaffold instead (14a–b, Scheme 5).^48^ In this case, the benzocrown substituents were able to simultaneously bind larger alkali metal cations (K^+^, Rb^+^, Cs^+^) in a 1 : 1 fashion in the *open* parallel form, while such a binding mode was not possible in the more rigid *closed* form. The binding behavior was studied by extraction of aqueous solutions of alkali metal picrates with the receptor in CH_2_Cl_2_. In this solvent, the PSS ratio (*open*/*closed*) obtained upon 313 nm irradiation was determined as 9 : 91, and the *open* isomer could be recovered using >480 nm light. The benzo-15-crown-5 derivative 14a was the most selective for extracting K^+^ and Rb^+^ ions, and the efficiency was the highest with the *open* form (Ex_open_/Ex_PSS_ = 2.1 and 4.8 for K^+^ and Rb^+^, respectively). With benzo-18-crown-6 motifs (14b), binding of Cs^+^ was improved and its extraction was 2.1 times more efficient with the *open* isomer than with the PSS_313_ mixture.^49^

In a separate study, the authors investigated the influence of K^+^ and Rb^+^ binding on the quantum yield for photocyclization of 14a.^50^ The thiophene rings can have a parallel or anti-parallel arrangement with respect to each other, and photocyclization is allowed only in the latter conformation according to the Woodward–Hoffmann rules.^5^ Yet, the parallel conformer is favored by complexation with alkali metal cations, for example, by addition of Rb^+^ the ratio parallel/anti-parallel changed from 1 : 1 to 6 : 1. This stabilization of the photochemically inactive conformer leads to a 10-fold decrease in photoisomerization quantum yield (from 0.21 for 14a alone to 0.02 in the presence of K^+^ and Rb^+^ perchlorate salts) as was measured in MeCN/CHCl_3_ (1 : 4).

### 3.2 Interaction with other substrates

This work was extended to the attachment of aza-crown ethers by Kawai,^51^ and a similar approach was taken by Takeshita *et al.* to control the binding of sugars *via* ester formation with boronic acid substituents.^52^ In addition, the group of Reinhoudt incorporated two β-cyclodextrin units to modulate binding of a sulfonated porphyrin guest.^53^ They obtained a 30 : 70 (*open*/*closed*) ratio at the PSS upon 313 nm irradiation and showed recovery of the original *open* isomer by irradiation with >460 nm light. The binding affinity for *meso*-tetrakis(4-sulfonatophenyl)porphyrin in water was found to be 35 times larger for the *open* than for the *closed* isomer and photoinduced guest liberation was demonstrated *in situ*. By incorporating a phenyl linker between the dithienylethene photoswitch and the β-cyclodextrin units, the PSS_313_ ratio was improved to 8 : 92 (*open*/*closed*), however, the difference in binding affinity was reduced to 8-fold.^54^ As porphyrins can act as photosensitizers to generate singlet oxygen, their reversible binding could potentially offer control of this process.^55^

**Fig. 5:**
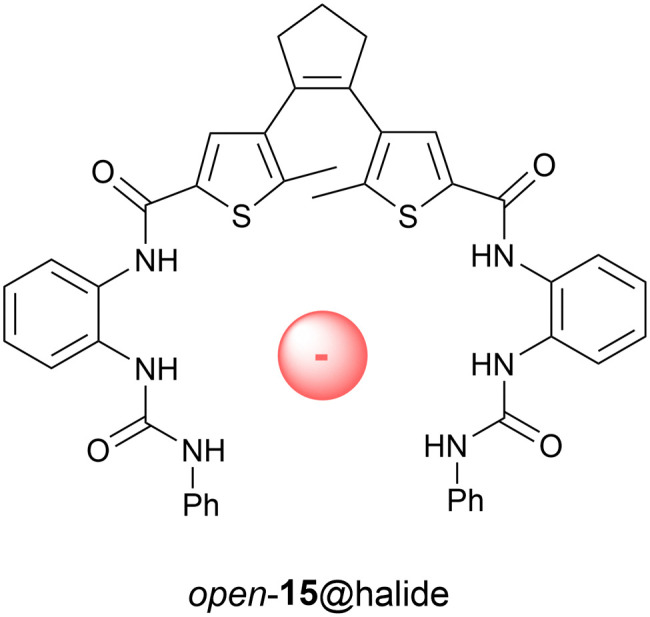
Dithienylethene-based receptor 15 able to bind chloride ions.

Towards photocontrol of anion binding affinity, Yin and Liu synthesized receptor 15 shown in Fig. 5.^56^ In this case, 302 nm irradiation gave the *closed* isomer in 34% yield and the *open* isomer was regenerated using >402 nm light. Among the halogen ions, the only noticeable difference in binding affinity was observed for chloride (*K*_a_ = 68 M^−1^ and 58 M^−1^ for *open* and *closed* forms in DMSO, respectively). According to energy minimization of the chloride-bound complexes by theoretical calculations, the open-ring isomer exists in the antiparallel conformation. Photoswitching (over multiple cycles) was shown feasible in the presence of chloride.

## 4. Stiff-stilbene

Where parent stilbene has been modified in an early stage with ethylenedioxy-substituents to extract alkali metal cations,^57^ and more recently with porphyrins to sequester fullerene,^58^ it is known to undergo undesired 6π-electrocyclization upon prolonged irradiation with UV light.^6*a*^ The fused five-membered ring analogue, *i.e.*, stiff-stilbene, is much more photostable and provides an excellent scaffold for synthesizing photoswitchable receptors.^6*b*^ Because of the rigid structure and the large change in geometry upon photoswitching, large differences in binding affinity between *trans* and *cis* isomers are generally observed. Furthermore, the energy barrier to thermal isomerization is very high, which allows to synthesize and examine both isomers independently. A drawback is that their operation requires high energy UV light, however, strategies to red-shift their excitation wavelength are currently being developed.^59^

### 4.1 Sterically overcrowded systems

The group of Shinmyozu was the first to consider stiff-stilbene as the scaffold of a photoswitchable receptor.^60^ They used a sterically crowded derivative with four methyl substituents in the five-membered rings close to the olefinic bond,^61^ and functionalized it with BINOL. The (*cis*/*trans*) isomer ratio upon 365 nm irradiation was estimated to be 86 : 14 in benzene and as 75 : 25 in acetonitrile. The reverse process, induced by irradiation with *ca.* 410 nm light, gave ratios of 23 : 77 and 9 : 91 in benzene and acetonitrile, respectively. The differences in binding properties for fluoride and chloride, studied in chloroform solution, were minor. However, the ^1^H NMR titration data for dihydrogen phosphate indicated a multistep equilibrium for the *trans* isomer, whereas the *cis* isomer formed the expected 1 : 1 complex.

**Scheme 6:**
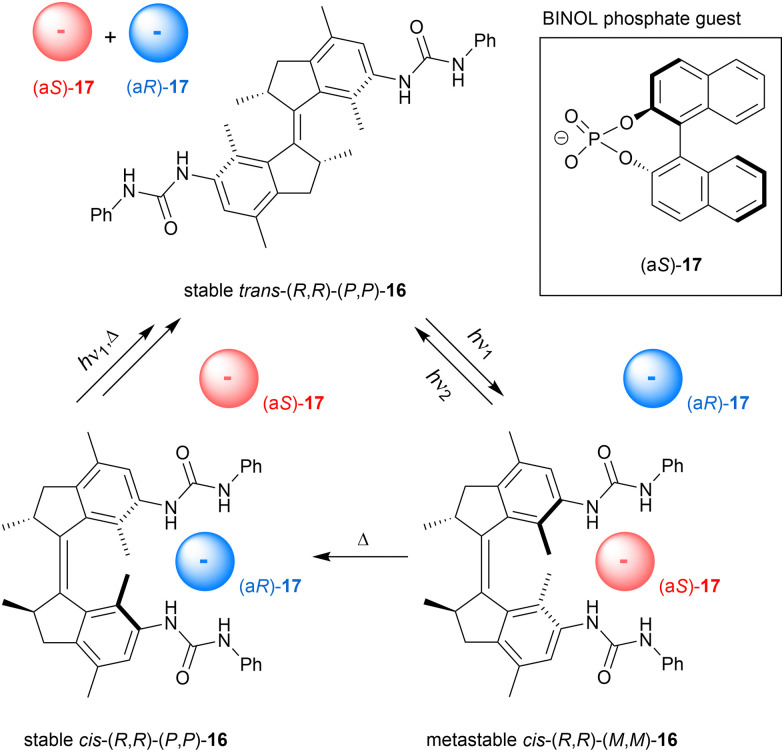
Three-state chirality-switchable anion receptor 16 based on molecular motor.

We started our journey in this field in 2014 by the attachment of two urea anion-binding motifs to stiff-stilbene derived molecular motor, which was used as a three-state switch in this case (16, Scheme 6).^62,63^ Starting with the stable *trans* isomer, 312 nm irradiation affords 80% of the metastable *cis* isomer, which can either equilibrate to the stable *cis* isomer by applying heat or quantitatively convert back to the stable *trans* isomer by 365 nm irradiation. When the stable *cis* isomer is irradiated with 312 nm light, the 80 : 20 metastable *cis*/stable *trans* PSS_312_ mixture is obtained directly because of the low thermal stability of the metastable *trans* intermediate. Where all the other photoswitchable tweezers are interconverted between two states, here three different states can thus be accessed at will by using light and heat.

The stable *cis* isomer proved selective for dihydrogen phosphate binding (*K*_a,1_ = 7.5 × 10^3^ M^−1^ in DMSO/0.5%H_2_O) and bound this anion much stronger than the stable *trans* isomer (*K*_*cis*_/*K*_*trans*_ = 57.7). Also acetate binding was substantial and displayed a significant difference in affinity between isomers (*K*_a,1(*cis*)_ = 1.3 × 10^3^ M^−1^ in DMSO/0.5%H_2_O and *K*_*cis*_/*K*_*trans*_ = 18.3). The highly distinct association constants for stable *cis* and *trans* isomers reflect their different binding modes: the former is able to tightly bind the oxo-anion by both urea substituents in a 1 : 1 stoichiometry, as supported by DFT geometry optimizations, while the latter can bind the anion only by a single urea substituent resulting in 1 : 2 complex formation upon saturation. Interestingly, binding of dihydrogen phosphate to metastable *cis*-16 was 3 times weaker than to stable *cis*-16 (*K*_a,1_ = 2.3 × 10^3^ M^−1^ in DMSO/0.5%H_2_O), which we ascribed to the slightly larger central dihedral angle compared to the stable *cis* isomer. Apparently, the positioning of the anion-binding substituents in the metastable *cis* form is therefore slightly less favored for cooperative binding than in the stable *cis* form. Owing to these multiple affinity differences, the amount of bound and unbound dihydrogen phosphate in solution could be regulated between three levels in a defined sequence of events, as was demonstrated *in situ* by a ^31^P NMR experiment.

It is important to note that the stable and metastable *cis* isomers have opposite helical chirality. By using the optically pure receptor, we were able to demonstrate light- and heat-controlled inversion of stereoselective binding of chiral BINOL phosphate guest 17 (Scheme 6).^64^ As mentioned earlier, 312 nm irradiation of the stable *cis* isomer directly affords a metastable *cis*/stable *trans* mixture in 80 : 20 ratio and by subsequently applying heat the former is converted to the starting isomer. Titration experiments with chiral guest 17 to stable *cis*-(*R*,*R*)-(*P*,*P*)-16 revealed a preference for the (a*R*)-enantiomer (*K*_R_/*K*_S_ = 4.2), which was the opposite for the metastable *cis*-(*R*,*R*)-(*M*,*M*)-16 (*K*_R_/*K*_S_ = 0.31). For the latter isomer, also an overall decrease in binding affinity was noted, similar to the observation with dihydrogen phosphate (*vide supra*). The enantioselectivity was improved by adding steric bulk to the chiral guest species, however, this went at the cost of binding affinity. As expected, binding of guest 17 to *trans*-(*R*,*R*)-(*P*,*P*)-16 was weak and moreover, did not show significant selectivity. This work was the first demonstration of dynamically-controlled stereoselective binding to a chiral receptor. In a recent study, Feringa ad Qu applied a similar concept to molecular motor embedded in a crown-ether, in which the enantioselective binding of a chiral ammonium guest could be inverted.^65^

### 4.2 Substrate-controlled (helical) isomerization

**Scheme 7:**
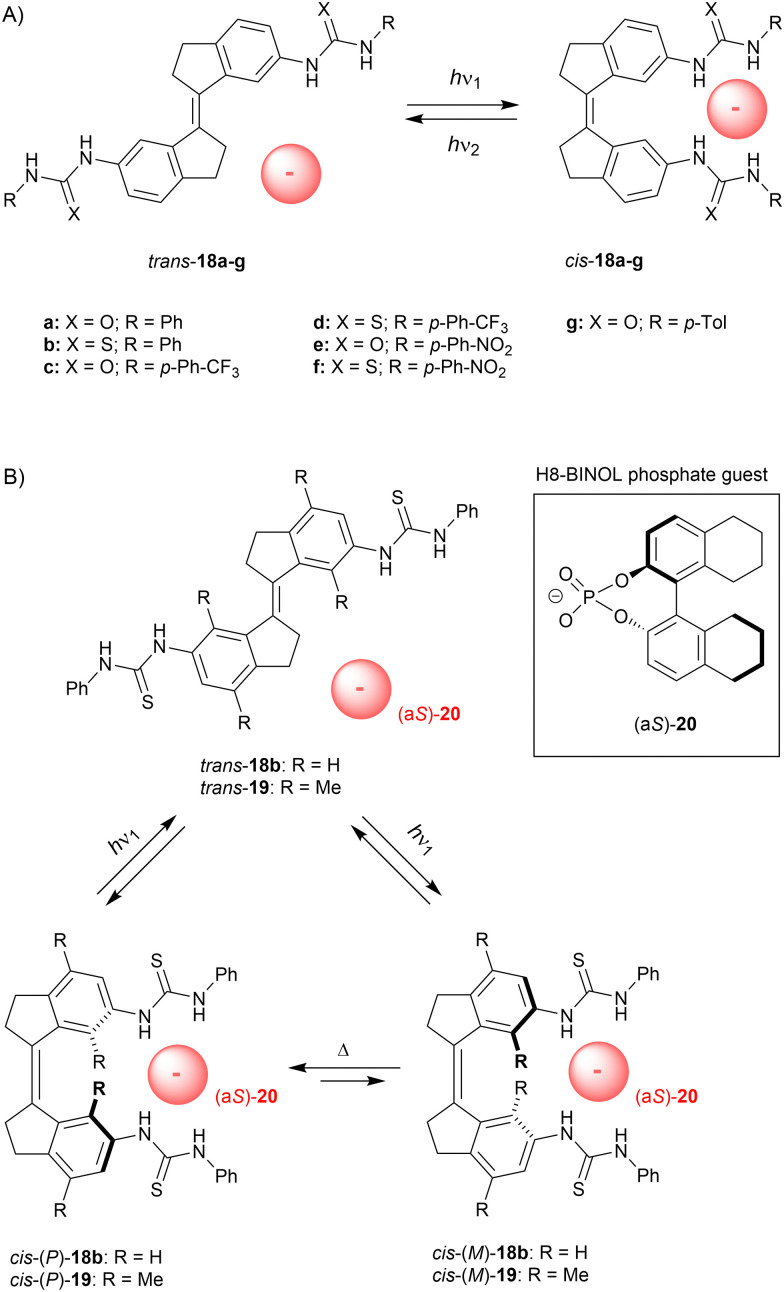
(A) Stiff-stilbene based anion receptors and (B) rotary motion induced by a chiral phosphate guest.

In later stage we simplified our design to non-methylated stiff-stilbene based receptor 18a (Scheme 7A),^66^ which can be operated with slightly longer irradiation wavelengths and exists as only two (stable) isomers. Now, *trans*-to-*cis* isomerization was induced using 365 nm light to give a (*cis*/*trans*) ratio of 51 : 49 at the PSS and the reverse isomerization process, which was triggered by 385 nm light, afforded a ratio of 7 : 93. Again, the *cis* isomer bound dihydrogen phosphate and acetate the strongest (*K*_a,1_ = 2.0 × 10^3^ M^−1^ and 1.4 × 10^3^ M^−1^ in DMSO/0.5%H_2_O, respectively), and much lower affinity constants were calculated for *trans*-18a (*K*_*cis*_/*K*_*trans*_ = 26.2 and 13.5, respectively). Photoisomerization was additionally studied in the presence of acetate, which seemed to slightly favor isomerization towards the *cis* isomer (*cis*/*trans* PSS_365_ = 52 : 48 and PSS_385_ = 12 : 88). In a later stage, Song and co-workers inserted a phenyl ring in between the stiff-stilbene scaffold and the urea groups, and reported similar photoisomerization and binding properties.^67^

Although the *trans* isomer is virtually planar, the *cis* isomer adopts *P* and *M* helical conformations. These interconvert very fast at rt (Δ^‡^*G*° = 16.7 kJ mol^−1^ as calculated by DFT) and the *cis* isomer therefore exists as racemate.^68^ Yet, we envisioned that binding of a chiral substrate would favor one of these helical isomers, such that where *trans*-to-*cis* isomerization would lead to equal formation of the *P*- and *M*-helical isomers, the backwards process would take place predominantly from one of them, resulting in net unidirectional rotation over the central double bond (see Scheme 7B). Helicity induction was indeed confirmed by CD spectroscopic titrations using the more soluble bis-thiourea derivative 18b and H8-BINOL phosphate guest 20, which has no spectral overlap with the stiff-stilbene receptor (>300 nm). Addition of (a*S*)-20 to *cis*-18b in dichloromethane gave rise to a positive signal in the CD spectrum and the exact opposite negative signal was observed upon addition of (a*R*)-20. The positive band was assigned to the *P*-helical isomer based on DFT calculations, which also confirmed that *cis*-(*P*)-18b⊂(a*S*)-20 is lower in energy than *cis*-(*M*)-18b⊂(a*S*)-20 by 5.6 kJ mol^−1^. At low temperature (−55 °C), these two possible diastereomeric complexes could be distinguished in the ^1^H NMR spectrum and were present in a 10 : 1 ratio.

Next, the *trans* isomer was irradiated with 365 nm light in presence of either (a*S*)-20 or (a*R*)-20 (2 equiv.), which led to the appearance of the same CD signal as was observed when the chiral guest was added to the *cis* isomer directly. Irradiation thus leads to generation of *cis*-18b and immediate induction of one of the helical forms. Interestingly, the PSS_365_ ratio (*cis*/*trans*) increased in presence of the guest from 58 : 42 to 76 : 24, but the quantum yield for *cis*-to-*trans* isomerization was not considerably altered (*Φ* = 18.2% and 20.1% without and with guest, respectively). Since the absorption of *trans*-18b and *cis*-18b is nearly the same at the irradiation wavelength, the difference in PSS_365_ ratio must stem from a lowering of the quantum yield for the backwards *cis*-to-*trans* isomerization. Whether this influence on the quantum yield as well as the enhancement of the PSS ratio should be ascribed to strong guest binding,^69^ electronic effects, or other factors, still needs to be investigated.

To exclude that enantioenrichment takes place already in the photochemical step, which could compromise unidirectionality, we later synthesized the more sterically crowded 19.^70^ For this compound, *P*/*M* helical inversion was much slower and, after addition of the chiral guest, the ratio between diastereomeric complexes could be monitored over time and changed from 1 : 1 to 1 : 1.27 after 3 days in DMSO, whereas in dichloromethane a ratio of 1 : 1.46 was reached in *ca.* 12 h. Also here, DFT calculations indicated that the (a*S*)-guest favors the (*P*)-helical receptor and the stability constants of *cis*-(*P*)-19⊂(a*S*)-20 and *cis*-(*M*)-19⊂(a*S*)-20 were estimated by a ^1^H NMR titration experiment as *K*_a_ = 4.6 × 10^2^ M^−1^ and 3.1 × 10^2^ M^−1^, respectively. Isomerization from the *trans* to the *cis* isomer was induced by 312 nm light to give a (*cis*/*trans*) ratio of 55 : 45 at the PSS, while the backwards process induced by 365 nm light afforded a ratio of 13 : 87. Both these ratios were slightly favored toward the *cis* isomer (∼5%) in the presence of the guest anion. Most importantly, a 1 : 1 ratio between diastereomeric complexes [*i.e.*, *cis*-(*P*)-19⊂(a*S*)-20 and *cis*-(*M*)-19⊂(a*S*)-20] was observed right after PSS_312_ was reached. This equimolar ratio illustrates that enantioenrichment solely occurs in the thermally activated step and thus, net unidirectional rotation was unequivocally established. It should be noted that other artificial molecular motors consist of an asymmetric molecular structure, or require a specific sequence of chemical transformations.^63*b*^ This supramolecularly-directed approach therefore represents a major breakthrough and will likely contribute to future development of more complex and sophisticated molecular machinery.

In these cases, the chiral substrate was thus intentionally used to exert control over helical isomerization (*i.e.*, *P*/*M* ratio). This control allows net unidirectional rotation over the central C=C bond when isomerized between *cis* and *trans* configurations. It is somewhat opposite to the concept described in the previous section, where light- and heat-controlled helical isomerization in a chiral bis-urea tweezer was used to invert enantioselective substrate binding. Overall, for these (thio)urea-appended stiff-stilbenes, the content of *cis* isomer in the PSS mixture only slightly increased when the anionic substrate was present, with the exception of H8-BINOL phosphate binding to 18b. It was shown here that the ∼20% increase in (*cis*/*trans*) PSS ratio stems from a reduced quantum yield for the *cis*-to-*trans* isomerization step. It should be noted that these studies were performed in dichloromethane, whereas in the other examples DMSO was used as the solvent. The underlying importance of binding strength, kinetics, as well as possible charge transfer requires further investigation.

### 4.3 Transport and diffusion

**Fig. 6:**
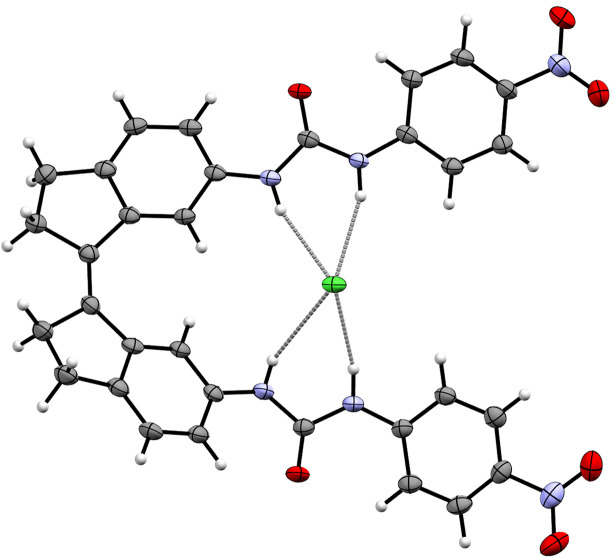
Displacement ellipsoid plot (50% probability level) of *cis*-18e⊂Cl^−^ (CCDC 2111230).^71^

In collaboration with the group of Gale, we studied the transmembrane transport properties of 18a–b as well as of *p*-trifluoromethyl- and *p*-nitro-substituted derivatives 18c–f (see Scheme 7A).^71^ Overall, 365 nm irradiation gave the highest conversion towards the *cis* isomers in case of the bis-thiourea compounds (47–52% *vs.* 35–51% for bis-thiourea and bis-urea, respectively), but at 385 nm irradiation the largest amount of *trans* isomer was recovered for the bis-ureas (93% for 18a, c*vs.* 74–83% for 18b, d). Furthermore, the PSS_365_ ratio of the *p*-nitro-substituted variants was not altered by subsequent irradiation with 385 nm light as the absorption spectra of *cis* and *trans* isomers (while being red-shifted) were highly similar. The binding affinity of chloride to all *cis* isomers, determined in DMSO/0.5%H_2_O, was found to be 5–6 times higher than to the respective *trans* isomers, and single crystal X-ray analysis of *cis*-18e⊂Cl^−^ showed the expected 1 : 1 complex with four NH⋯Cl^−^ hydrogen bonds (Fig. 6).

The chloride transport activity of 18a–f was first evaluated using an HPTS assay, in which the *cis* isomers proved much more effective than the *trans* isomers. The highest activity was found for *cis*-18f (EC_50_ = 0.002 mol% to lipid) and in case of 18e, the *cis* isomer was 568-fold more active than the *trans* isomer. Here, the difference in transport activity is thus two orders of magnitude larger than that in binding affinity, highlighting that the latter is not the determinant factor for the former. Additional mechanistic studies using cation co-transporters in various liposomal transport assays revealed that phenyl(thio)ureas 18a–b are highly selective for transporting Cl^−^ (over H^+^ and OH^−^), *i.e.*, they are electrogenic uniporters. Next, the compounds were used to depolarize liposomes having a pre-established K^+^ concentration gradient, for which *cis*-18b turned out to be the most efficient. Apart from reversibly controlling transport activity *in situ* by alternating irradiation wavelengths, we demonstrated membrane depolarization (and concomitant built-up of a chloride gradient) by light. Hence, to a certain degree this system emulates the function of light-sensitive halorhodopsin and anion channel rhodopsin proteins, which are able to respectively polarize and depolarize cells by the selective flow of chloride ions.

The tolyl-derivative 18g (Scheme 7A) was synthesized in an effort to study the effect of (light-controlled) anion binding on the receptor's diffusion rate.^72^ The *p*-tolyl methyl ^1^H NMR signal was convenient to track in DOSY-NMR experiments. The *trans* isomer diffuses slightly slower (by 7%) than the *cis* isomer because of its more elongated structure. It was found by the group of Beves that dihydrogen phosphate is able to assemble in DMSO solution (∼50 mM) into oligomers by anti-electrostatic hydrogen bonding. Due to the formation of receptor/oligomer complexes in presence of excess dihydrogen phosphate, the diffusion rate of *trans*-18g and *cis*-18g decreased by 33% and 26%, respectively. Since this decrease is larger for the *trans* isomer, the overall change in diffusion upon photoinduced *cis*/*trans* isomerization is enhanced from 7% in absence to 16% in presence of dihydrogen phosphate, where the latter equals a 70% change in effective volume. With this anion-enhanced change in diffusion upon photoisomerization, the next step would be to drive directional motion of the receptor toward light or concentration gradients (*cf.* chemotactic behavior).

## 5. Hemi-thioindigo

Hemi-thioindigo consists of a stilbene fragment that is fused with a thioindigo fragment.^7^ It is worth noting that thioindigo – having ethylene glycol side groups – was used earlier to control metal binding by Irie *et al.*^73^ The key advantage of these molecular switches is that they can be operated using visible light instead of harmful UV light. On the other hand, even though the geometrical change upon isomerization is large, its functionalization to obtain a tweezer-type receptor is less straightforward than with azobenzene or stiff-stilbene.

**Scheme 8:**
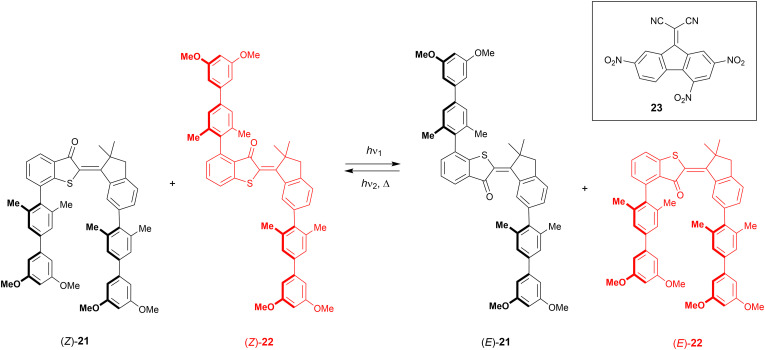
Complementary hemi-thioindigo derived molecular tweezers 21–22 used in the translocation of electron-deficient guest 23.

Following their work on a hemi-thioindigo based helical receptor,^74^ the group of Dube reported molecular tweezers 21 and 22 (Scheme 8).^75^ The photoswitchable scaffold contains electron-rich biphenyl substituents, which in *Z*-21 are pointing in the same direction, while in *Z*-22 they are pointing away from each other. In the former compound, the initial geometry is ideal for binding an electron-deficient aromatic guest, which would be released upon isomerization to the *E* isomer, while for the latter, the exact opposite effect is expected. Both compounds had similar absorption profiles and by 435 nm irradiation, the *E* isomers were produced in 63% and 86% for 21 and 22, respectively. By using 530 nm light, the reverse isomerization took place resulting in the recovery of 84% of *Z*-21 and 80% of *Z*-22. Also in the dark, the *E* isomers thermally equilibrated to the respective *Z* isomers, albeit not quantitatively, and a *Z*-enriched mixture was obtained.

The electron-poor 9-(dicyanomethylene)-2,4,7-trinitrofluorene (23) was chosen as guest and binding studies were performed in chloroform solution at −20 °C, to avoid ^1^H NMR signal broadening observed at rt. Whereas with *Z*-22 and *E*-21 virtually no binding was observed, *Z*-21 and *E*-22 strongly bound the guest in a 1 : 1 stoichiometry with association constants of *K*_a_ = 2.3 × 10^3^ M^−1^ and 1.2 × 10^4^ M^−1^, respectively. As the isomerization of both compounds is induced by the same wavelengths, they can be switched simultaneously from the *Z* to the *E* isomers and *vice versa* using a single irradiation wavelength, allowing guest translocation. Such translocation was demonstrated by pre-irradiating a 1 : 1 mixture of 21 and 22 with either 435 nm or 530 nm light, until the PSS was reached, and then the guest (0.6 equiv.) was added, after which the solutions were irradiated again. After irradiation at 435 nm, the guest was primarily bound by *E*-22 and upon 530 nm irradiation, most of it was transferred to *Z*-21. A single wavelength can thus be used to concurrently trigger the isomerization of two complementary tweezers, which are able to pass a guest molecule between them.

In a subsequent study, 21 was oxidized to obtain its sulfoxide derivative, which had improved photoswitching properties, thermal stability, as well as binding affinity.^76^ Although the overall absorption was blue-shifted relative to the parent compound, now 83% of the *E* isomer could be produced using 405 nm light and 80% of the *Z* isomer was recovered by irradiation with 470 nm light. Again, the *E* form did practically not bind electron-deficient aromatic guest, but the *Z* form did with an elevated binding constant of *K*_a_ = 4.1 × 10^3^ M^−1^ in chloroform at −20 °C (*vs. K*_a_ = 2.3 × 10^3^ M^−1^ for *Z*-21).

## 6. Conclusions

The equipment of molecular photoswitches with two binding entities offers a simple means to reversibly control substrate binding by light. In one of the photoaddressable states, the binding motifs are in close proximity to simultaneously bind a guest species (high affinity), while in the other they are too far apart from each other to attain the same 1 : 1 binding mode (low affinity). Obviously, this method is the most successful when the used photoswitch is rigid and undergoes a pronounced change in geometry upon isomerization (*e.g.*, in case of azobenzene and stiff-stilbene). Nevertheless, substantial differences in binding affinity have been achieved in some cases with dithienylethene-based receptors. In terms of applications, the first studies in which photoswitchable tweezers serve either as extractants or as carriers (to mediate transmembrane transport) have been reported, but there is still much to explore. Important aspects such as partitioning between organic and aqueous phase as well as visible-light-excitation need to be properly addressed before real-world applications come in sight.

Where in all studies reversible photoswitching is successfully demonstrated and the isomers of the receptor are shown to possess distinct binding affinity, the influence of guest binding on the isomerization properties is not always discussed. It is important though, since it has been found that the switching behavior can change significantly in presence of the substrate. With respect to thermal *cis*-to-*trans* isomerization of azobenzene, rate acceleration/deceleration has been ascribed to a “tying” effect in stable sandwich-type 1 : 1 complexes as well as to electron-donating and -withdrawing effects, where it can also be a combination of the two. Effects on the photoisomerization of dithienylethene have been related to (de)stabilization of the anti-parallel conformer, which is photochemically inactive. For azobenzene and stiff-stilbene, the substrate-binding effects on photoisomerization behavior are more difficult to explain. While it has been frequently found that the stronger binding isomer is favored in the PSS mixture when the substrate is present, care should be taken with the interpretation of this result. As the PSS ratio depends on both molar absorptivity and isomerization quantum yield, measurement of the latter would provide a better insight into the role of substrate binding. In one specific case, we found that the quantum yield for *cis*-to-*trans* isomerization of stiff-stilbene bis-thiourea was reduced in the presence of H8-BINOL phosphate, but whether this should be ascribed to tight guest binding or to electronic effects still needs to be fully clarified. Additionally, it was shown that a chiral substrate can be used to influence helical isomerization, which allowed to direct net unidirectional rotary motion.

Photoswitchable molecular tweezers are interesting from both perspectives. On the one hand, they provide excellent control of binding affinity offering promising applications in extraction technology and transmembrane transport. On the other hand, they are well suited to investigate the effects of supramolecular binding on isomerization behavior, which will open the way for the development of light-driven molecular switches and machines that are controlled/gated by chemical stimuli. We are looking forward to such new and exciting developments.

## Conflicts of interest

There are no conflicts to declare.

## Supplementary Material
